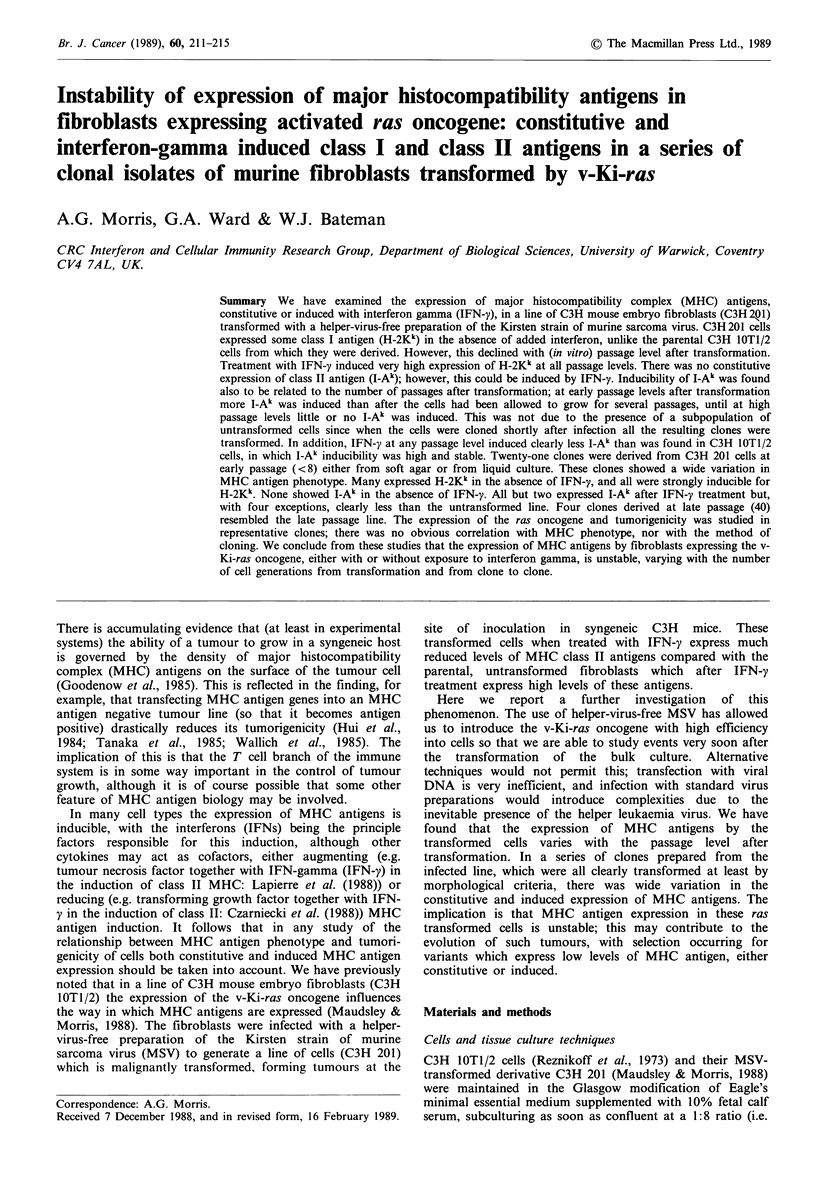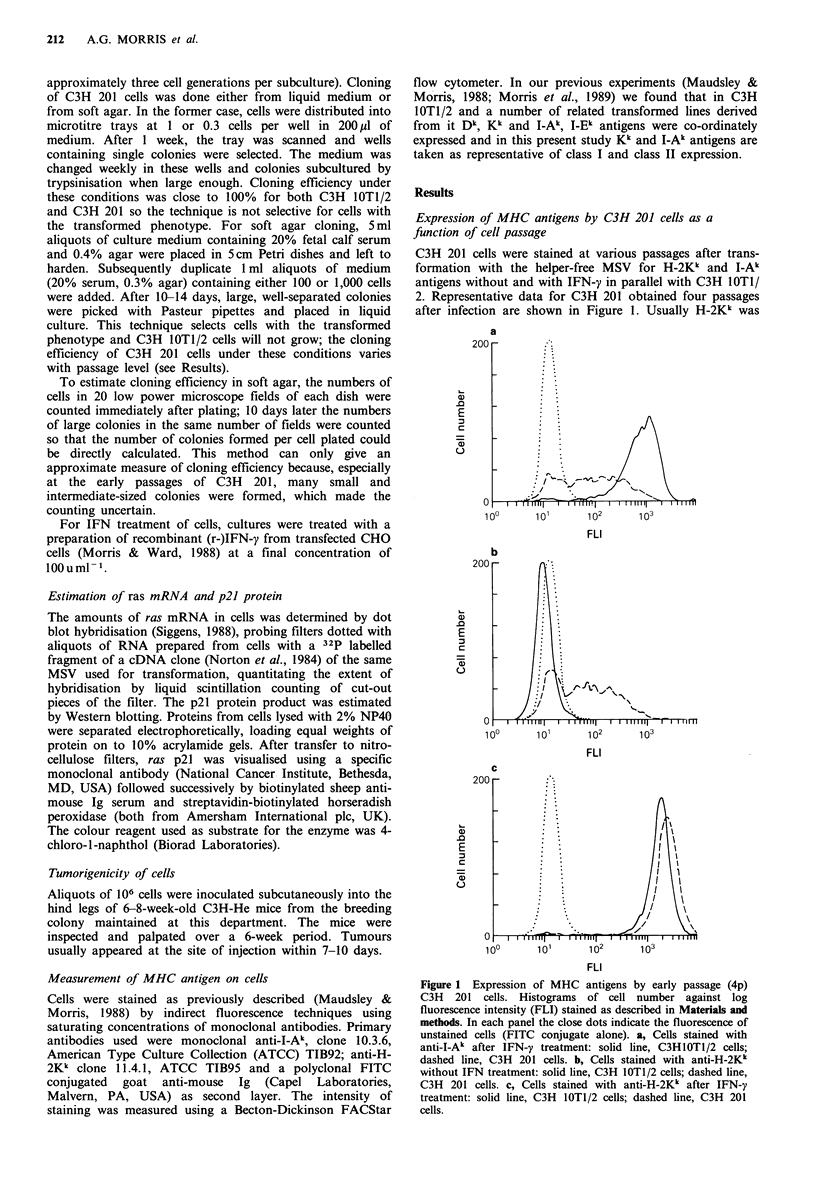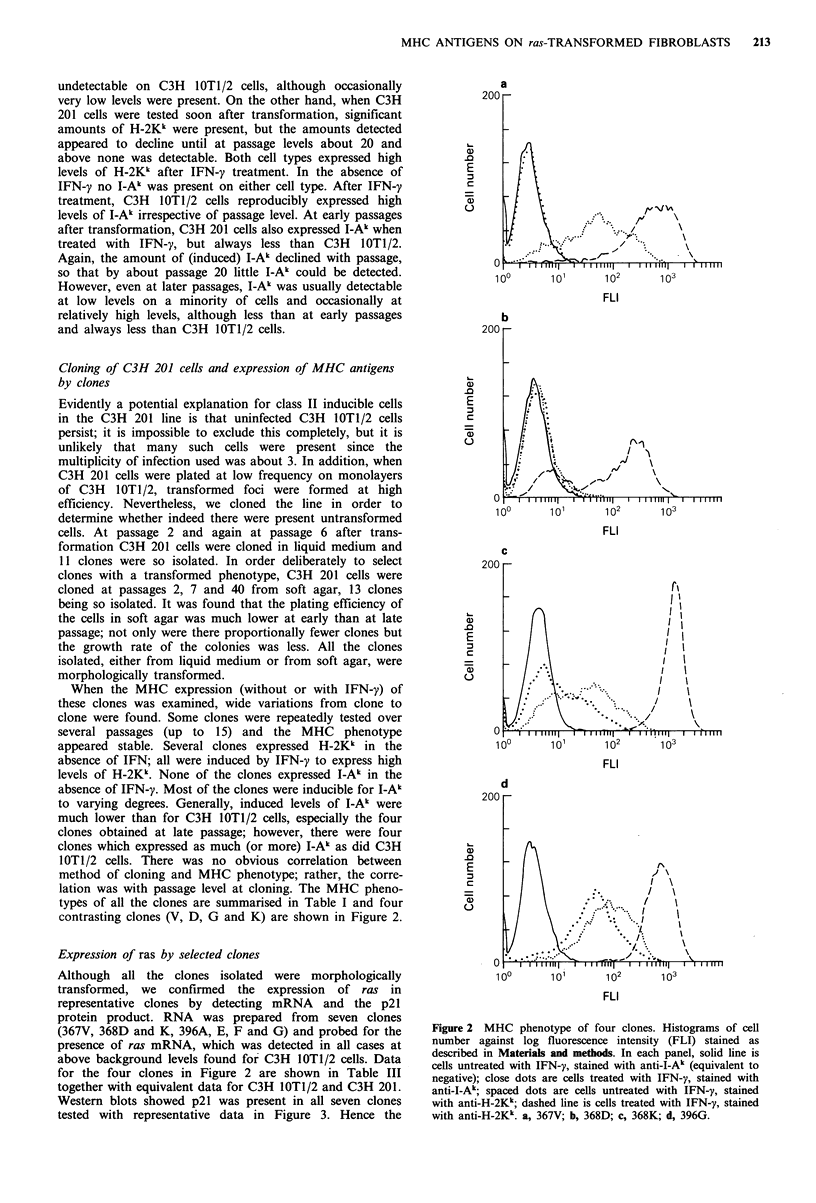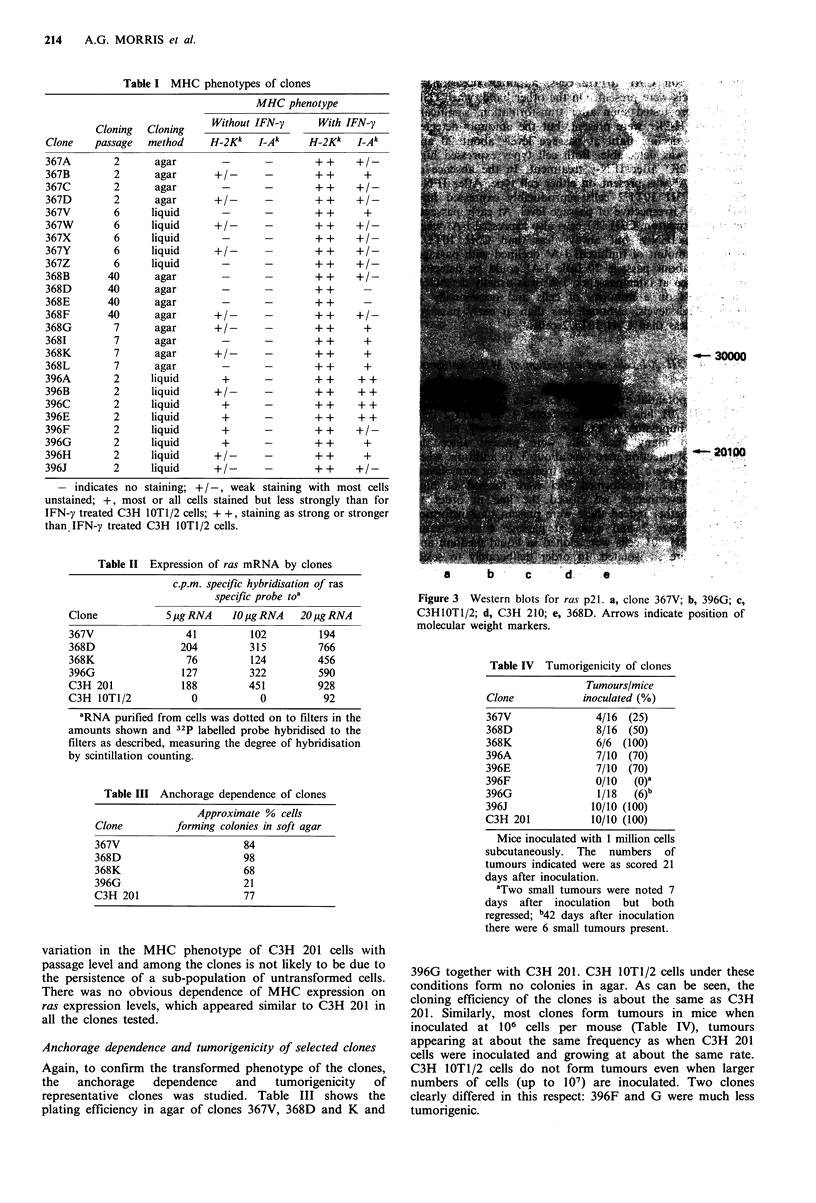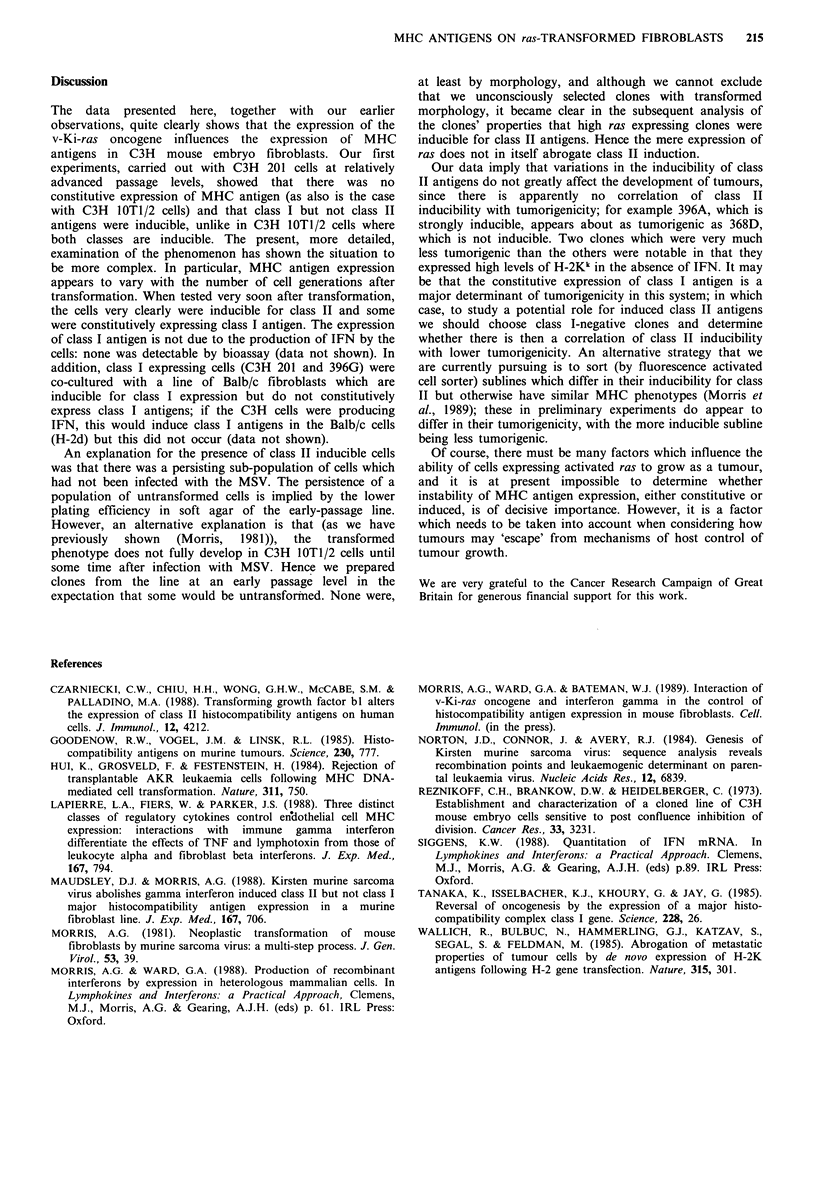# Instability of expression of major histocompatibility antigens in fibroblasts expressing activated ras oncogene: constitutive and interferon-gamma induced class I and class II antigens in a series of clonal isolates of murine fibroblasts transformed by v-Ki-ras.

**DOI:** 10.1038/bjc.1989.253

**Published:** 1989-08

**Authors:** A. G. Morris, G. A. Ward, W. J. Bateman

**Affiliations:** CRC Interferon and Cellular Immunity Research Group, University of Warwick, Coventry, UK.

## Abstract

**Images:**


Br. J. Cancer (1989), 60, 211–215 © The Macmillan Press Ltd., 1989

### Instability of expression of major histocompatibility antigens in fibroblasts expressing activated *ras* oncogene: constitutive and interferon-gamma induced class I and class II antigens in a series of clonal isolates of murine fibroblasts transformed by v-Ki-*ras*

A.G. Morris, G.A. Ward & W.J. Bateman

*CRC Interferon and Cellular Immunity Research Group, Department of Biological Sciences, University of Warwick, Coventry CV4 7AL, UK.*

**Summary** We have examined the expression of major histocompatibility complex (MHC) antigens, constitutive or induced with interferon gamma (IFN-$\gamma$), in a line of C3H mouse embryo fibroblasts (C3H 201) transformed with a helper-virus-free preparation of the Kirsten strain of murine sarcoma virus. C3H 201 cells expressed some class I antigen (H-2K$^k$) in the absence of added interferon, unlike the parental C3H 10T1/2 cells from which they were derived. However, this declined with (*in vitro*) passage level after transformation. Treatment with IFN-$\gamma$ induced very high expression of H-2K$^k$ at all passage levels. There was no constitutive expression of class II antigen (I-A$^k$); however, this could be induced by IFN-$\gamma$. Inducibility of I-A$^k$ was found also to be related to the number of passages after transformation; at early passage levels after transformation more I-A$^k$ was induced than after the cells had been allowed to grow for several passages, until at high passage levels little or no I-A$^k$ was induced. This was not due to the presence of a subpopulation of untransformed cells since when the cells were cloned shortly after infection all the resulting clones were transformed. In addition, IFN-$\gamma$ at any passage level induced clearly less I-A$^k$ than was found in C3H 10T1/2 cells, in which I-A$^k$ inducibility was high and stable. Twenty-one clones were derived from C3H 201 cells at early passage (<8) either from soft agar or from liquid culture. These clones showed a wide variation in MHC antigen phenotype. Many expressed H-2K$^k$ in the absence of IFN-$\gamma$, and all were strongly inducible for H-2K$^k$. None showed I-A$^k$ in the absence of IFN-$\gamma$. All but two expressed I-A$^k$ after IFN-$\gamma$ treatment but, with four exceptions, clearly less than the untransformed line. Four clones derived at late passage (40) resembled the late passage line. The expression of the *ras* oncogene and tumorigenicity was studied in representative clones; there was no obvious correlation with MHC phenotype, nor with the method of cloning. We conclude from these studies that the expression of MHC antigens by fibroblasts expressing the v-Ki-*ras* oncogene, either with or without exposure to interferon gamma, is unstable, varying with the number of cell generations from transformation and from clone to clone.

There is accumulating evidence that (at least in experimental systems) the ability of a tumour to grow in a syngeneic host is governed by the density of major histocompatibility complex (MHC) antigens on the surface of the tumour cell (Goodenow *et al.*, 1985). This is reflected in the finding, for example, that transfecting MHC antigen genes into an MHC antigen negative tumour line (so that it becomes antigen positive) drastically reduces its tumorigenicity (Hui *et al.*, 1984; Tanaka *et al.*, 1985; Wallich *et al.*, 1985). The implication of this is that the *T* cell branch of the immune system is in some way important in the control of tumour growth, although it is of course possible that some other feature of MHC antigen biology may be involved.

In many cell types the expression of MHC antigens is inducible, with the interferons (IFNs) being the principle factors responsible for this induction, although other cytokines may act as cofactors, either augmenting (e.g. tumour necrosis factor together with IFN-gamma (IFN-$\gamma$) in the induction of class II MHC: Lapierre *et al.* (1988)) or reducing (e.g. transforming growth factor together with IFN-$\gamma$ in the induction of class II: Czarniecki *et al.* (1988)) MHC antigen induction. It follows that in any study of the relationship between MHC antigen phenotype and tumorigenicity of cells both constitutive and induced MHC antigen expression should be taken into account. We have previously noted that in a line of C3H mouse embryo fibroblasts (C3H 10T1/2) the expression of the v-Ki-*ras* oncogene influences the way in which MHC antigens are expressed (Maudsley & Morris, 1988). The fibroblasts were infected with a helper-virus-free preparation of the Kirsten strain of murine sarcoma virus (MSV) to generate a line of cells (C3H 201) which is malignantly transformed, forming tumours at the site of inoculation in syngeneic C3H mice. These transformed cells when treated with IFN-$\gamma$ express much reduced levels of MHC class II antigens compared with the parental, untransformed fibroblasts which after IFN-$\gamma$ treatment express high levels of these antigens.

Here we report a further investigation of this phenomenon. The use of helper-virus-free MSV has allowed us to introduce the v-Ki-*ras* oncogene with high efficiency into cells so that we are able to study events very soon after the transformation of the bulk culture. Alternative techniques would not permit this; transfection with viral DNA is very inefficient, and infection with standard virus preparations would introduce complexities due to the inevitable presence of the helper leukaemia virus. We have found that the expression of MHC antigens by the transformed cells varies with the passage level after transformation. In a series of clones prepared from the infected line, which were all clearly transformed at least by morphological criteria, there was wide variation in the constitutive and induced expression of MHC antigens. The implication is that MHC antigen expression in these *ras* transformed cells is unstable; this may contribute to the evolution of such tumours, with selection occurring for variants which express low levels of MHC antigen, either constitutive or induced.

### Materials and methods

### *Cells and tissue culture techniques*

C3H 10T1/2 cells (Reznikoff *et al.*, 1973) and their MSV-transformed derivative C3H 201 (Maudsley & Morris, 1988) were maintained in the Glasgow modification of Eagle's minimal essential medium supplemented with 10% fetal calf serum, subculturing as soon as confluent at a 1:8 ratio (i.e.

Correspondence: A.G. Morris.
Received 7 December 1988, and in revised form, 16 February 1989.

approximately three cell generations per subculture). Cloning of C3H 201 cells was done either from liquid medium or from soft agar. In the former case, cells were distributed into microtitre trays at 1 or 0.3 cells per well in 200 μl of medium. After 1 week, the tray was scanned and wells containing single colonies were selected. The medium was changed weekly in these wells and colonies subcultured by trypsinisation when large enough. Cloning efficiency under these conditions was close to 100% for both C3H 10T1/2 and C3H 201 so the technique is not selective for cells with the transformed phenotype. For soft agar cloning, 5 ml aliquots of culture medium containing 20% fetal calf serum and 0.4% agar were placed in 5 cm Petri dishes and left to harden. Subsequently duplicate 1 ml aliquots of medium (20% serum, 0.3% agar) containing either 100 or 1,000 cells were added. After 10–14 days, large, well-separated colonies were picked with Pasteur pipettes and placed in liquid culture. This technique selects cells with the transformed phenotype and C3H 10T1/2 cells will not grow; the cloning efficiency of C3H 201 cells under these conditions varies with passage level (see Results).

To estimate cloning efficiency in soft agar, the numbers of cells in 20 low power microscope fields of each dish were counted immediately after plating; 10 days later the numbers of large colonies in the same number of fields were counted so that the number of colonies formed per cell plated could be directly calculated. This method can only give an approximate measure of cloning efficiency because, especially at the early passages of C3H 201, many small and intermediate-sized colonies were formed, which made the counting uncertain.

For IFN treatment of cells, cultures were treated with a preparation of recombinant (r-)IFN-γ from transfected CHO cells (Morris & Ward, 1988) at a final concentration of 100 u ml$^{-1}$.

*Estimation of* ras *mRNA and p21 protein*

The amounts of *ras* mRNA in cells was determined by dot blot hybridisation (Siggens, 1988), probing filters dotted with aliquots of RNA prepared from cells with a $^{32}P$ labelled fragment of a cDNA clone (Norton *et al.*, 1984) of the same MSV used for transformation, quantitating the extent of hybridisation by liquid scintillation counting of cut-out pieces of the filter. The p21 protein product was estimated by Western blotting. Proteins from cells lysed with 2% NP40 were separated electrophoretically, loading equal weights of protein on to 10% acrylamide gels. After transfer to nitrocellulose filters, *ras* p21 was visualised using a specific monoclonal antibody (National Cancer Institute, Bethesda, MD, USA) followed successively by biotinylated sheep anti-mouse Ig serum and streptavidin-biotinylated horseradish peroxidase (both from Amersham International plc, UK). The colour reagent used as substrate for the enzyme was 4-chloro-1-naphthol (Biorad Laboratories).

*Tumorigenicity of cells*

Aliquots of $10^6$ cells were inoculated subcutaneously into the hind legs of 6–8-week-old C3H-He mice from the breeding colony maintained at this department. The mice were inspected and palpated over a 6-week period. Tumours usually appeared at the site of injection within 7–10 days.

*Measurement of MHC antigen on cells*

Cells were stained as previously described (Maudsley & Morris, 1988) by indirect fluorescence techniques using saturating concentrations of monoclonal antibodies. Primary antibodies used were monoclonal anti-I-A$^k$, clone 10.3.6, American Type Culture Collection (ATCC) TIB92; anti-H-2K$^k$ clone 11.4.1, ATCC TIB95 and a polyclonal FITC conjugated goat anti-mouse Ig (Capel Laboratories, Malvern, PA, USA) as second layer. The intensity of staining was measured using a Becton-Dickinson FACStar flow cytometer. In our previous experiments (Maudsley & Morris, 1988; Morris *et al.*, 1989) we found that in C3H 10T1/2 and a number of related transformed lines derived from it D$^k$, K$^k$ and I-A$^k$, I-E$^k$ antigens were co-ordinately expressed and in this present study K$^k$ and I-A$^k$ antigens are taken as representative of class I and class II expression.

### Results

*Expression of MHC antigens by C3H 201 cells as a function of cell passage*

C3H 201 cells were stained at various passages after transformation with the helper-free MSV for H-2K$^k$ and I-A$^k$ antigens without and with IFN-γ in parallel with C3H 10T1/2. Representative data for C3H 201 obtained four passages after infection are shown in Figure 1. Usually H-2K$^k$ was

**Figure 1** Expression of MHC antigens by early passage (4p) C3H 201 cells. Histograms of cell number against log fluorescence intensity (FLI) stained as described in **Materials and methods**. In each panel the close dots indicate the fluorescence of unstained cells (FITC conjugate alone). **a**, Cells stained with anti-I-A$^k$ after IFN-γ treatment: solid line, C3H10T1/2 cells; dashed line, C3H 201 cells. **b**, Cells stained with anti-H-2K$^k$ without IFN treatment: solid line, C3H 10T1/2 cells; dashed line, C3H 201 cells. **c**, Cells stained with anti-H-2K$^k$ after IFN-γ treatment: solid line, C3H 10T1/2 cells; dashed line, C3H 201 cells.

undetectable on C3H 10T1/2 cells, although occasionally very low levels were present. On the other hand, when C3H 201 cells were tested soon after transformation, significant amounts of H-2K$^k$ were present, but the amounts detected appeared to decline until at passage levels about 20 and above none was detectable. Both cell types expressed high levels of H-2K$^k$ after IFN-$\gamma$ treatment. In the absence of IFN-$\gamma$ no I-A$^k$ was present on either cell type. After IFN-$\gamma$ treatment, C3H 10T1/2 cells reproducibly expressed high levels of I-A$^k$ irrespective of passage level. At early passages after transformation, C3H 201 cells also expressed I-A$^k$ when treated with IFN-$\gamma$, but always less than C3H 10T1/2. Again, the amount of (induced) I-A$^k$ declined with passage, so that by about passage 20 little I-A$^k$ could be detected. However, even at later passages, I-A$^k$ was usually detectable at low levels on a minority of cells and occasionally at relatively high levels, although less than at early passages and always less than C3H 10T1/2 cells.

### *Cloning of C3H 201 cells and expression of MHC antigens by clones*

Evidently a potential explanation for class II inducible cells in the C3H 201 line is that uninfected C3H 10T1/2 cells persist; it is impossible to exclude this completely, but it is unlikely that many such cells were present since the multiplicity of infection used was about 3. In addition, when C3H 201 cells were plated at low frequency on monolayers of C3H 10T1/2, transformed foci were formed at high efficiency. Nevertheless, we cloned the line in order to determine whether indeed there were present untransformed cells. At passage 2 and again at passage 6 after transformation C3H 201 cells were cloned in liquid medium and 11 clones were so isolated. In order deliberately to select clones with a transformed phenotype, C3H 201 cells were cloned at passages 2, 7 and 40 from soft agar, 13 clones being so isolated. It was found that the plating efficiency of the cells in soft agar was much lower at early than at late passage; not only were there proportionally fewer clones but the growth rate of the colonies was less. All the clones isolated, either from liquid medium or from soft agar, were morphologically transformed.

When the MHC expression (without or with IFN-$\gamma$) of these clones was examined, wide variations from clone to clone were found. Some clones were repeatedly tested over several passages (up to 15) and the MHC phenotype appeared stable. Several clones expressed H-2K$^k$ in the absence of IFN; all were induced by IFN-$\gamma$ to express high levels of H-2K$^k$. None of the clones expressed I-A$^k$ in the absence of IFN-$\gamma$. Most of the clones were inducible for I-A$^k$ to varying degrees. Generally, induced levels of I-A$^k$ were much lower than for C3H 10T1/2 cells, especially the four clones obtained at late passage; however, there were four clones which expressed as much (or more) I-A$^k$ as did C3H 10T1/2 cells. There was no obvious correlation between method of cloning and MHC phenotype; rather, the correlation was with passage level at cloning. The MHC phenotypes of all the clones are summarised in Table I and four contrasting clones (V, D, G and K) are shown in Figure 2.

### *Expression of* ras *by selected clones*

Although all the clones isolated were morphologically transformed, we confirmed the expression of *ras* in representative clones by detecting mRNA and the p21 protein product. RNA was prepared from seven clones (367V, 368D and K, 396A, E, F and G) and probed for the presence of *ras* mRNA, which was detected in all cases at above background levels found for C3H 10T1/2 cells. Data for the four clones in Figure 2 are shown in Table III together with equivalent data for C3H 10T1/2 and C3H 201. Western blots showed p21 was present in all seven clones tested with representative data in Figure 3. Hence the

**Figure 2** MHC phenotype of four clones. Histograms of cell number against log fluorescence intensity (FLI) stained as described in **Materials and methods**. In each panel, solid line is cells untreated with IFN-$\gamma$, stained with anti-I-A$^k$ (equivalent to negative); close dots are cells treated with IFN-$\gamma$, stained with anti-I-A$^k$; spaced dots are cells untreated with IFN-$\gamma$, stained with anti-H-2K$^k$; dashed line is cells treated with IFN-$\gamma$, stained with anti-H-2K$^k$. **a**, 367V; **b**, 368D; **c**, 368K; **d**, 396G.

**Table I** MHC phenotypes of clones

| Clone | Cloning passage | Cloning method | MHC phenotype: Without IFN-$\gamma$ H-2K$^k$ | Without IFN-$\gamma$ I-A$^k$ | With IFN-$\gamma$ H-2K$^k$ | With IFN-$\gamma$ I-A$^k$ |
|---|---|---|---|---|---|---|
| 367A | 2 | agar | − | − | ++ | +/− |
| 367B | 2 | agar | +/− | − | ++ | + |
| 367C | 2 | agar | − | − | ++ | +/− |
| 367D | 2 | agar | +/− | − | ++ | +/− |
| 367V | 6 | liquid | − | − | ++ | + |
| 367W | 6 | liquid | +/− | − | ++ | +/− |
| 367X | 6 | liquid | − | − | ++ | +/− |
| 367Y | 6 | liquid | +/− | − | ++ | +/− |
| 367Z | 6 | liquid | − | − | ++ | +/− |
| 368B | 40 | agar | − | − | ++ | +/− |
| 368D | 40 | agar | − | − | ++ | − |
| 368E | 40 | agar | − | − | ++ | − |
| 368F | 40 | agar | +/− | − | ++ | +/− |
| 368G | 7 | agar | +/− | − | ++ | + |
| 368I | 7 | agar | − | − | ++ | + |
| 368K | 7 | agar | +/− | − | ++ | + |
| 368L | 7 | agar | − | − | ++ | + |
| 396A | 2 | liquid | + | − | ++ | ++ |
| 396B | 2 | liquid | +/− | − | ++ | ++ |
| 396C | 2 | liquid | + | − | ++ | ++ |
| 396E | 2 | liquid | + | − | ++ | ++ |
| 396F | 2 | liquid | + | − | ++ | +/− |
| 396G | 2 | liquid | + | − | ++ | + |
| 396H | 2 | liquid | +/− | − | ++ | + |
| 396J | 2 | liquid | +/− | − | ++ | +/− |

− indicates no staining; +/−, weak staining with most cells unstained; +, most or all cells stained but less strongly than for IFN-$\gamma$ treated C3H 10T1/2 cells; ++, staining as strong or stronger than IFN-$\gamma$ treated C3H 10T1/2 cells.

**Table II** Expression of *ras* mRNA by clones

| Clone | c.p.m. specific hybridisation of ras specific probe to[a]: 5 μg RNA | 10 μg RNA | 20 μg RNA |
|---|---|---|---|
| 367V | 41 | 102 | 194 |
| 368D | 204 | 315 | 766 |
| 368K | 76 | 124 | 456 |
| 396G | 127 | 322 | 590 |
| C3H 201 | 188 | 451 | 928 |
| C3H 10T1/2 | 0 | 0 | 92 |

[a]RNA purified from cells was dotted on to filters in the amounts shown and $^{32}P$ labelled probe hybridised to the filters as described, measuring the degree of hybridisation by scintillation counting.

**Table III** Anchorage dependence of clones

| Clone | Approximate % cells forming colonies in soft agar |
|---|---|
| 367V | 84 |
| 368D | 98 |
| 368K | 68 |
| 396G | 21 |
| C3H 201 | 77 |

**Figure 3** Western blots for *ras* p21. **a**, clone 367V; **b**, 396G; **c**, C3H10T1/2; **d**, C3H 210; **e**, 368D. Arrows indicate position of molecular weight markers.

**Table IV** Tumorigenicity of clones

| Clone | Tumours/mice inoculated (%) |
|---|---|
| 367V | 4/16 (25) |
| 368D | 8/16 (50) |
| 368K | 6/6 (100) |
| 396A | 7/10 (70) |
| 396E | 7/10 (70) |
| 396F | 0/10 (0)[a] |
| 396G | 1/18 (6)[b] |
| 396J | 10/10 (100) |
| C3H 201 | 10/10 (100) |

Mice inoculated with 1 million cells subcutaneously. The numbers of tumours indicated were as scored 21 days after inoculation.

[a]Two small tumours were noted 7 days after inoculation but both regressed; [b]42 days after inoculation there were 6 small tumours present.

variation in the MHC phenotype of C3H 201 cells with passage level and among the clones is not likely to be due to the persistence of a sub-population of untransformed cells. There was no obvious dependence of MHC expression on *ras* expression levels, which appeared similar to C3H 201 in all the clones tested.

*Anchorage dependence and tumorigenicity of selected clones*

Again, to confirm the transformed phenotype of the clones, the anchorage dependence and tumorigenicity of representative clones was studied. Table III shows the plating efficiency in agar of clones 367V, 368D and K and 396G together with C3H 201. C3H 10T1/2 cells under these conditions form no colonies in agar. As can be seen, the cloning efficiency of the clones is about the same as C3H 201. Similarly, most clones form tumours in mice when inoculated at $10^6$ cells per mouse (Table IV), tumours appearing at about the same frequency as when C3H 201 cells were inoculated and growing at about the same rate. C3H 10T1/2 cells do not form tumours even when larger numbers of cells (up to $10^7$) are inoculated. Two clones clearly differed in this respect: 396F and G were much less tumorigenic.

### Discussion

The data presented here, together with our earlier observations, quite clearly shows that the expression of the v-Ki-*ras* oncogene influences the expression of MHC antigens in C3H mouse embryo fibroblasts. Our first experiments, carried out with C3H 201 cells at relatively advanced passage levels, showed that there was no constitutive expression of MHC antigen (as also is the case with C3H 10T1/2 cells) and that class I but not class II antigens were inducible, unlike in C3H 10T1/2 cells where both classes are inducible. The present, more detailed, examination of the phenomenon has shown the situation to be more complex. In particular, MHC antigen expression appears to vary with the number of cell generations after transformation. When tested very soon after transformation, the cells very clearly were inducible for class II and some were constitutively expressing class I antigen. The expression of class I antigen is not due to the production of IFN by the cells: none was detectable by bioassay (data not shown). In addition, class I expressing cells (C3H 201 and 396G) were co-cultured with a line of Balb/c fibroblasts which are inducible for class I expression but do not constitutively express class I antigens; if the C3H cells were producing IFN, this would induce class I antigens in the Balb/c cells (H-2d) but this did not occur (data not shown).

An explanation for the presence of class II inducible cells was that there was a persisting sub-population of cells which had not been infected with the MSV. The persistence of a population of untransformed cells is implied by the lower plating efficiency in soft agar of the early-passage line. However, an alternative explanation is that (as we have previously shown (Morris, 1981)), the transformed phenotype does not fully develop in C3H 10T1/2 cells until some time after infection with MSV. Hence we prepared clones from the line at an early passage level in the expectation that some would be untransformed. None were, at least by morphology, and although we cannot exclude that we unconsciously selected clones with transformed morphology, it became clear in the subsequent analysis of the clones' properties that high *ras* expressing clones were inducible for class II antigens. Hence the mere expression of *ras* does not in itself abrogate class II induction.

Our data imply that variations in the inducibility of class II antigens do not greatly affect the development of tumours, since there is apparently no correlation of class II inducibility with tumorigenicity; for example 396A, which is strongly inducible, appears about as tumorigenic as 368D, which is not inducible. Two clones which were very much less tumorigenic than the others were notable in that they expressed high levels of H-2K$^k$ in the absence of IFN. It may be that the constitutive expression of class I antigen is a major determinant of tumorigenicity in this system; in which case, to study a potential role for induced class II antigens we should choose class I-negative clones and determine whether there is then a correlation of class II inducibility with lower tumorigenicity. An alternative strategy that we are currently pursuing is to sort (by fluorescence activated cell sorter) sublines which differ in their inducibility for class II but otherwise have similar MHC phenotypes (Morris *et al.*, 1989); these in preliminary experiments do appear to differ in their tumorigenicity, with the more inducible subline being less tumorigenic.

Of course, there must be many factors which influence the ability of cells expressing activated *ras* to grow as a tumour, and it is at present impossible to determine whether instability of MHC antigen expression, either constitutive or induced, is of decisive importance. However, it is a factor which needs to be taken into account when considering how tumours may 'escape' from mechanisms of host control of tumour growth.

We are very grateful to the Cancer Research Campaign of Great Britain for generous financial support for this work.

### References

CZARNIECKI, C.W., CHIU, H.H., WONG, G.H.W., McCABE, S.M. & PALLADINO, M.A. (1988). Transforming growth factor b1 alters the expression of class II histocompatibility antigens on human cells. *J. Immunol.*, **12,** 4212.

GOODENOW, R.W., VOGEL, J.M. & LINSK, R.L. (1985). Histocompatibility antigens on murine tumours. *Science*, **230,** 777.

HUI, K., GROSVELD, F. & FESTENSTEIN, H. (1984). Rejection of transplantable AKR leukaemia cells following MHC DNA-mediated cell transformation. *Nature*, **311,** 750.

LAPIERRE, L.A., FIERS, W. & PARKER, J.S. (1988). Three distinct classes of regulatory cytokines control endothelial cell MHC expression: interactions with immune gamma interferon differentiate the effects of TNF and lymphotoxin from those of leukocyte alpha and fibroblast beta interferons. *J. Exp. Med.*, **167,** 794.

MAUDSLEY, D.J. & MORRIS, A.G. (1988). Kirsten murine sarcoma virus abolishes gamma interferon induced class II but not class I major histocompatibility antigen expression in a murine fibroblast line. *J. Exp. Med.*, **167,** 706.

MORRIS, A.G. (1981). Neoplastic transformation of mouse fibroblasts by murine sarcoma virus: a multi-step process. *J. Gen. Virol.*, **53,** 39.

MORRIS, A.G. & WARD, G.A. (1988). Production of recombinant interferons by expression in heterologous mammalian cells. In *Lymphokines and Interferons: a Practical Approach*, Clemens, M.J., Morris, A.G. & Gearing, A.J.H. (eds) p. 61. IRL Press: Oxford.

MORRIS, A.G., WARD, G.A. & BATEMAN, W.J. (1989). Interaction of v-Ki-*ras* oncogene and interferon gamma in the control of histocompatibility antigen expression in mouse fibroblasts. *Cell. Immunol.* (in the press).

NORTON, J.D., CONNOR, J. & AVERY, R.J. (1984). Genesis of Kirsten murine sarcoma virus: sequence analysis reveals recombination points and leukaemogenic determinant on parental leukaemia virus. *Nucleic Acids Res.*, **12,** 6839.

REZNIKOFF, C.H., BRANKOW, D.W. & HEIDELBERGER, C. (1973). Establishment and characterization of a cloned line of C3H mouse embryo cells sensitive to post confluence inhibition of division. *Cancer Res.*, **33,** 3231.

SIGGENS, K.W. (1988). Quantitation of IFN mRNA. In *Lymphokines and Interferons: a Practical Approach.* Clemens, M.J., Morris, A.G. & Gearing, A.J.H. (eds) p.89. IRL Press: Oxford.

TANAKA, K., ISSELBACHER, K.J., KHOURY, G. & JAY, G. (1985). Reversal of oncogenesis by the expression of a major histocompatibility complex class I gene. *Science*, **228,** 26.

WALLICH, R., BULBUC, N., HAMMERLING, G.J., KATZAV, S., SEGAL, S. & FELDMAN, M. (1985). Abrogation of metastatic properties of tumour cells by *de novo* expression of H-2K antigens following H-2 gene transfection. *Nature*, **315,** 301.